# No Clear Clustering Dysbiosis from Salivary Microbiota Analysis by Long Sequencing Reads in Patients Affected by Oral Squamous Cell Carcinoma: A Single Center Study

**DOI:** 10.3390/cancers15174211

**Published:** 2023-08-22

**Authors:** Rodolfo Mauceri, Martina Coppini, Davide Vacca, Giorgio Bertolazzi, Valeria Cancila, Claudio Tripodo, Giuseppina Campisi

**Affiliations:** 1 Department of Surgical, Oncological and Oral Sciences, University of Palermo, 90127 Palermo, Italy; rodolfo.mauceri@unipa.it (R.M.); davide.vacca@unipa.it (D.V.); giuseppina.campisi@policlinico.pa.it (G.C.); 2 Unit of Oral Medicine and Dentistry for Frail Patients, Department of Rehabilitation, Fragility and Continuity of Care, University Hospital Palermo, 90127 Palermo, Italy; 3 Department of Biomedical and Dental Sciences and Morphofunctional Imaging, University of Messina, 90100 Messina, Italy; 4 Tumor Immunology Unit, Department of Sciences for Health Promotion and Mother-Child Care “G. D’Alessandro”, University of Palermo, 90127 Palermo, Italy; giorgio.bertolazzi@unipa.it (G.B.); valeria.cancila@unipa.it (V.C.); claudio.tripodo@unipa.it (C.T.); 5 Department of Economics, Business and Statistics, University of Palermo, 90128 Palermo, Italy

**Keywords:** microbiota, Next Generation Sequencing (NGS), metagenomics, silico estimation, microbial composition, squamous cell carcinoma of head and neck, OSCC

## Abstract

**Simple Summary:**

The present study aimed to investigate the salivary microbiota composition employing for the first time in the literature the Oxford Nanopore Technology in patients affected by oral squamous cell carcinoma (OSCC). Unstimulated saliva samples from 24 patients affected by OSCC and 7 patients free from OSCC were collected and analyzed. In the OSCC group, 13 patients were males and 11 females with a mean age of 65.5 ± 13.9 years; in the control group, 5 patients were males and 2 females with a mean age of 51.4 ± 19.2 years. Regarding the salivary microbiota composition, *Prevotella*, *Chlamydia*, *Tissierellia*, *Calothrix*, *Leotiomycetes*, *Firmicutes* and *Zetaproteobacteria* were the most abundant microorganisms detected in OSCC patients. If the association between the alteration of salivary microbiota composition and OSCC onset was confirmed, it could have significant implications in the prevention strategy and in follow-up visits.

**Abstract:**

Background: Advancements in DNA sequencing technology have facilitated the assessment of the connection between the oral microbiome and various diseases. The aim of the present study was to investigate the salivary microbiota composition employing for the first time in the literature the Oxford Nanopore Technology in patients affected by oral squamous cell carcinoma (OSCC). Methods: Unstimulated saliva samples of 31 patients were collected (24 OSCC patients and 7 controls). DNA was extracted using the QIAamp DNA Blood Kit and metagenomic long sequencing reads were performed using the MinION device. Results: In the OSCC group, 13 were males and 11 were females, with a mean age of 65.5 ± 13.9 years; in the control group, 5 were males and 2 were females, with a mean age of 51.4 ± 19.2 years. The border of the tongue was the most affected OSCC site. The microorganisms predominantly detected in OSCC patients were *Prevotella*, *Chlamydia*, *Tissierellia*, *Calothrix*, *Leotiomycetes*, *Firmicutes* and *Zetaproteobacteria*. Conclusions: This study confirmed the predominance of periodontopathic bacteria in the salivary microbiome in the OSCC group. If a direct correlation between oral dysbiosis and OSCC onset was proven, it could lead to new prevention strategies and early diagnostic tools.

## 1. Introduction

The oral cavity is a complex and dynamic ecosystem responsive to environmental and biological changes, in which resides over 700 species of bacteria, fungi, viruses, and protozoa [1].

In 1988 the term “microbiome” was introduced to define “a characteristic microbial community occupying a reasonably well-defined habitat which has distinct physico-chemical properties” [2].

The balance of the oral microbiome is maintained by a continuous interplay with the host. Variations in this state of equilibrium are termed “dysbiosis”, which may allow pathogens to cause diseases, such as caries and periodontitis [3]. 

Through rapidly developing DNA sequencing methodology (i.e., Next Generation Sequencing, NGS) and analytical techniques, in recent years, several studies have explored the association between the human microbiome and different types of disease, including cancer [4,5,6]. For example, *Helicobacter pylori* has been associated with gastric cancer, *Salmonella typhi* with gallbladder cancer, and *Fusobacterium nucleatum* with colon cancer [7,8,9]. Among the NGS technologies, the Oxford Nanopore Technology (ONT) is a new technology generation able to perform very long sequencing reads and to sequence native DNA and RNA without the requirement of the pre-amplification of published targets, so enabling both to discover any genome present in the sample and to eliminate possible bias PCR-related.

To date, the relationship between the oral microbiome and oral squamous cell carcinoma (OSCC) has not yet been definitively demonstrated. OSCC is a multifactorial disease that arises from both host genetics and environmental factors. Despite most of the OSCC risk factors are well-known (e.g., tobacco smoking, alcohol consumption), the incidence of OSCC still is high and the prognosis of patients affected by this cancer remains unsatisfactory [10,11,12]. 

The oral microbiome is characterized by some properties that make it unique and equally difficult to study. Indeed, oral microbiota is a complex dynamic ecological community conditioned by continuous changes in the availability of oxygen, nutrients, and the pH of saliva, since it contains very distinct niches adhering to various surfaces [13]. According to the Human Oral Microbiome Database, only approximately 57% of the oral bacterial species were identified, 13% were cultivated but they remain nameless, and 30% are neither isolable nor replicable in cultures microbiological [14].

The aim of the present study was to investigate the salivary microbiota composition employing for the first time in the literature the ONT in patients affected by OSCC.

## 2. Materials and Methods

### 2.1. Ethics Statement

The study protocol conformed to the ethical guidelines of the 1964 Declaration of Helsinki and its later amendments or comparable ethical standards. The study was approved by the institutional review board of the “Paolo Giaccone” Policlinico University Hospital in Palermo (Italy) (approval #11/2020). Written informed consent was obtained from all participants involved in the study. The study reported was assessed following the STROBE guidelines for observational studies.

### 2.2. Population Recruitment

The subjects were unselectively and consecutively recruited from the Unit of Oral Medicine at the “Paolo Giaccone” Policlinico University Hospital in Palermo (Italy), from December 2020 to May 2022. 

The patients were selected based on the following inclusion criteria:

i. Age older than 18 years;
ii. Patients affected by OSCC confirmed by pathological findings;
iii. Ability to provide informed consent.

Patients were excluded if they were pregnant or nursing, received antibiotics or periodontal therapy in the previous 3 months, if they were affected by severe periodontitis, if they had any immunocompromising conditions, or if they did receive any previous surgical treatment, radiotherapy, and/or chemotherapy.

To confirm the diagnosis of OSCC, a histopathological examination was undertaken. After local anaesthesia, an incisional biopsy was performed. The section from one sample was fixed in formalin solution and sent to the pathology laboratory for histopathological OSCC diagnosis. 

As a control, patients affected by oral disease different from OSCC and healthy patients were enrolled.

### 2.3. Outcome Measures

For each patient, the following data were recorded: demographic data, localization of OSCC lesion according to International Classification of Diseases (ICD, 10th revision), TNM stage (version 7.0) according to the guidelines of American Joint Committee on Cancer (AJCC) and the International Union Against Cancer (UICC), smoking habits, alcohol drinking and traumatic risk factors (i.e., incongruous prostheses or sharp cusp). Based on the TNM stage, the patients were classified as early (stage I and II) or advanced stage (stage III and IV).

### 2.4. Sample Collection

Non-stimulated saliva was obtained in the morning from each participant using an established protocol. Patients were asked to refrain from eating, drinking, or smoking for at least two hours prior to sample collection, and to rinse their mouths with saline solution for 60 s. Saliva was allowed to accumulate in the floor of the mouth and then was collected in sterile Falcon tubes and stored frozen at −80 °C immediately until ready to use.

### 2.5. DNA Extraction

From each sample has been extracted DNA using the QIAamp DNA Blood Kit (Qiagen, Cat No 51183, Hilden, Germany), following the QIAamp^®^ DNA Mini and Blood Mini protocol—Blood or Body fluid spin section.

Briefly, each falcon tube was centrifuged for 30–40 min at 300× *g* to pellet the lactescent, like-mucus phase and to remove the supernatant. Next, the pellet was resuspended in 400 µL of 1× PBS pH 7.4, homogenized by pipetting, and transferred in new 2 mL Eppendorf tubes. Next, any solution was mixed with 40 µL of Qiagen proteinase k and 400 µL di AL buffer, then incubated at 56 °C for 16 to 24 h, until the pellet was dissolved completely. Then, 8 µL of Qiagen Rnase A was added to each sample at a concentration of 100 ng/µL and then it was mixed by inversion at least 3 times and was incubated for 2 min at room temperature. Next, 400 µL of pure ethanol was added to each sample, and after it has been well mixed by pipetting, all mixture was filtered through the QIAamp Mini spin column. Subsequently, in the spin columns were applied 500 µL of both AW1 and AW2 buffer was for a total of 2 washes. In the elution step, each QIAamp Mini spin column was placed in a new clean 1.5 mL microcentrifuge collection tube and 70 µL di Tris-EDTA pH 8 was added directly onto the membrane. Each sample was incubated for 5 min at room temperature and successively centrifuged for 1 min at 20,000× *g* to recover the DNA. The elution step was repeated another time, recovering the DNA, and centrifuging in the same tube.

### 2.6. Samples Sequencing 

From DNA samples, we prepared metagenomic sequencing libraries with a Rapid PCR Barcoding Kit (SQK-RPB004—RPB_9059_v1_revL_14Aug2019) and then ran on the MinION device (ONT, Oxford, UK). We basecalled the output (i.e., converted the sequencing device output into nucleic acid base sequences) with the MinKNOW software v20.06.4 (Oxford Nanopore Technologies Ltd., Oxford, UK) (https://community.nanoporetech.com/technical_documents/minknow-techdoc/v/mitd_5000_v1_revah_16may2016/introduction-to-minknow, accessed on 16 May 2022) and used the ONT platform, EPI2ME, for quality control, species identification [What’s In My Pot (WIMP) pipeline]. DNA initial concentration was 5 ng per sample, in 3 μL of nuclease-free water. For the DNA tagmentation, 1 µL fragmentation mix (FRM) was added for each sample, which was incubated at 30 °C and then at 80 °C for 1 min per step, in a thermocycler. Next, 4 ng of the tegmented DNA was added to a mixture that included 1 μL of Rapid Barcode Primer at 10 µM, 25 μL of LongAmp^®^ Hot Start Taq 2× Master Mix (NEB cat No. M0287), and Nuclease-free water, up to a final volume of 50 µL. PCR profile included an initial denaturation of 3 min at 95 °C; 14 cycles of 15 s at 95 °C, 15 s at 56 °C, 6 min at 65 °C; and a final extension step of 6 min at 65 °C with a Hold at 4 °C. The amplified DNA was cleaned up with 30 µL of Agencourt AMPure XP beads (Beckman coulter, Munich, Germany) at 1× concentration, incubated in a rotator mixer for 10 min, and washed twice on a magnetic rack, with 200 µL of freshly made 70% ethanol. Next, after having to remove the ethanol and air drying the pellet, it was eluted in 10 μL of freshly T50 Buffer, made with 1% of Tris-HCl 1 M pH 8, 5% of NaCl 1 M, and 94% of Nuclease-free water; and then 1 µL per sample was quantified by Qubit™ fluorometer (Thermo Scientific: Waltham, MA, USA). To determine the average length of the library in each sample, 1 µL was analyzed in 2100 Bioanalyzer Instrument, model G2939B using Agilent DNA 12000 protocol (Part. Number 5067-1508), according to manufacturers’ instructions. The NEBioCalculator v1.15.0 web tool (https://nebiocalculator.neb.com/#!/dsdnaamt, accessed on 15 May 2022) was used to convert ng of the sample into fmol, and barcoded DNA was then pooled into a 1.5-mL microcentrifuge tube, at the final concentration of 100 fmol in 10 μL of T50 buffer. The rapid Adapter ligation step was performed by mixing the pooled barcoded sample with 1 µL di Rapid Adapter buffer (Oxford Nanopore Technologies Ltd., Oxford, UK.; SQK-PBK004) and incubating for 5 min at room temperature. The priming and loading step was conducted following the protocol instructions. Sequencing was performed on ONT MinION flow cell (FLO-MIN106 R9 Version) connected to an Mk1B device (ONT Ltd., Oxford, UK.; MIN-101B). 

The sequencing was run up to 48 h, using nanopore software MinKNOW v20.06.4, in live basecall mode with default parameters, so that it uses neural networks to directly translate and barcode the raw signals Fast5 file in barcoded fastq format. All fastq files were deposited in the NCBI SRA database under PRJxxxxxxxx. The data set was analyzed by cloud-based analysis WIMP application from the EPI2ME platform (Oxford Nanopore Technologies Ltd., Oxford, UK), which is based on Centrifuge which assigns taxonomy by comparing read sequences against the NCBI reference database [15]. 

### 2.7. Bioinformatics and Statistical Analysis

We used the tax_name function of the taximize R package to retrieve the class and the family names from the NCBI database [16]. The absolute frequencies of the organism correspond to the WIMP reads, and the absolute frequencies of classes have been calculated summing the reads of the organisms that belong to the same class. To consider the environmental contaminants that could occur during the sequencing, we have associated each human sample with a control experiment (white run), and we have analyzed the control experiments using WIMP. In this way we have identified the possible environmental contaminants related to each sample run. The absolute organism reads of each control experiment have been subtracted from the organism reads of the respective human sample. For hierarchical clustering analysis of the patients, the Bray Curtis was used to calculate the distance among patients and the Ward-D2 aggregation method was used for building the dendrogram through the R package hclust. We used the bootstrap *t*-test to compare the average absolute frequencies among patient groups. All statistical analyses have been performed using R statistical software v4.2.3 (http://www.R-project.org) [17].

## 3. Results

This section may be divided into subheadings. It should provide a concise and precise description of the experimental results, their interpretation, as well as the experimental conclusions that can be drawn.

### 3.1. Characteristics of Study Participants

The “sputum” samples were collected from 31 patients: 24 were affected by OSCC and 7 were free from OSCC. Among the last one, 3 patients were affected by oral potentially malignant disorders (control disease group) and 4 were healthy patients (control health group). The main characteristics of patients enrolled in the study were described in Table 1. 

In the OSCC group, 13 patients were males (13/24; 54.2%) and 11 were females (11/24; 45.8%), with a mean age of 65.5 ± 13.9 years (range 32–88 years). With respect to the OSCC site, the border of the tongue was the most affected site (10/24; 41.7%), followed by buccal mucosa (6/24; 25%), floor of the mouth (3/24; 12.5%), lower gingiva (3/24; 12.5%), upper gingiva (1/24; 4.1%), and hard palate (1/24; 4.1%). 

According to the TNM staging classification, OSCC cases were classified as follows: 7 patients with stage I (7/24; 29.2%), 6 patients with stage II (6/24; 25%), 5 patients with stage III (5/24; 20.8%), and 6 patients with stage IV (6/24; 25%). Based on the TNM stage, 13 patients were classified as early (13/24; 54.2%), and 11 were classified as advanced stage (11/24; 45.8%). Regarding habits of OSCC patients, 7 patients were smokers (7/24; 29.2%), among them, and 3 were both smokers and drinkers (3/24; 12.5%); 3 were former smokers (3/24; 12.5%). Regarding the potential mechanical trauma, clinical examination revealed the presence of incongruous prostheses in 4 patients (4/24; 16.7%).

No data about the missing teeth and decayed teeth were available. Regarding the dentures, 6 patients were wearing dental prostheses, of which 4 were patients affected by OSCC and 2 were healthy patients.

### 3.2. Microbiota Composition 

In the current study, the taxonomy of the salivary samples was analyzed through cloud-based analysis WIMP application from the EPI2ME platform.

**Figure 1:**
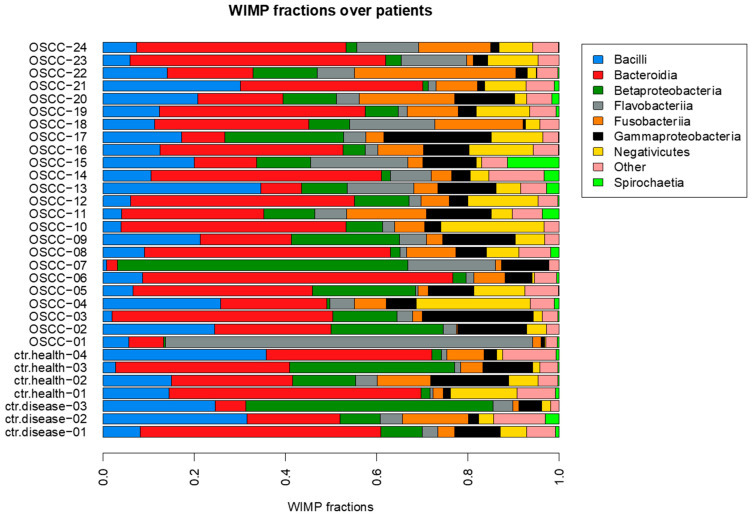
Relative frequencies of organism classes over patients.

We used the WIMP software v20.06.4 to obtain an in-silico estimation of the organism read counts over samples (relative frequencies in Figure 1, absolute frequencies in Supplementary Figure S1, Table S1) [18]. Then we compared the average counts between OSCC and control groups to identify the microorganisms whose counts are different on average.

As previously reported in the literature, the ONT Technology confirmed the prevalence of periodontal bacteria in patients affected by OSCC (i.e., *Prevotella*) [19]. In detail, in this study was observed that *Prevotella melaninogenica*, *Prevotella intermedia* and *Prevotella jejuni* were the highest expressed genes in the OSCC group.

Then we compared the average counts between OSCC and control groups to identify the microorganism classes whose counts are different on average. To cope with the low sample size, we used a non-parametric testing approach, based on the bootstrap *t*-test, after excluding microorganism classes present in less than 5 samples and with a total count lower than 5 [20]. Sixteen microorganism classes are statistically significant (*p*-value < 0.05). Indeed, in the OSCC group a significant increase in *Chlamydiia*, *Tissierellia*, *Calothrix*, *Leotiomycetes*, *Firmicutes*, and *Zetaproteobacteria*, and a decrease in *Saccharibacteria* was observed (Supplementary Table S2). Despite those classes being statistically significant, the Benjamini-Hochberg adjusted p-values are greater than the 5% threshold due to the low statistical power. 

**Figure 2:**
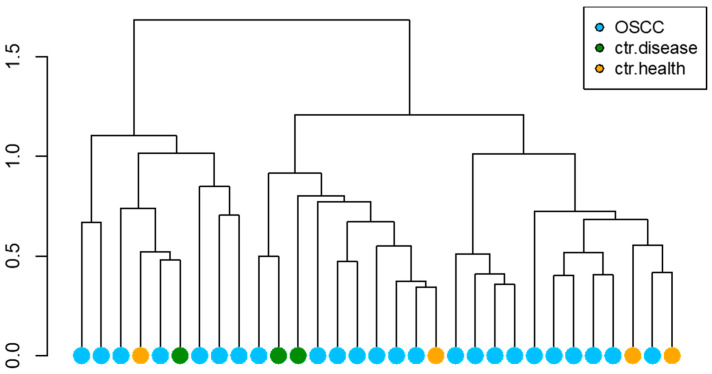
Hierarchical clustering over patients.

The hierarchical cluster analysis performed on the read counts does not show a clear clustering behavior of OSCC patients (Figure 2). 

**Figure 3:**
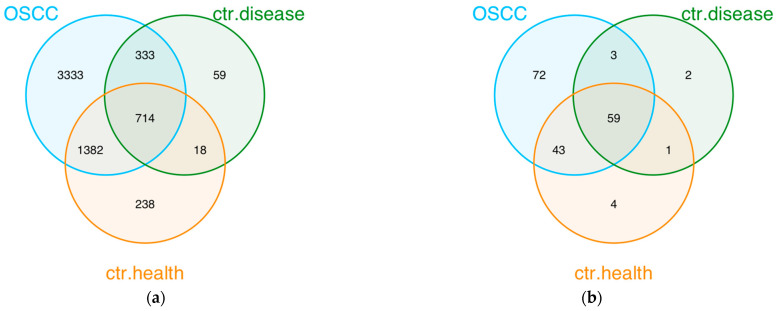
Organisms and class overlaps among groups. (**a**) Organisms overlaps among groups. (**b**) Class overlaps among groups.

A Venn diagram was used to illustrate the distinct and shared microorganisms and classes between groups. From this it follows that 714 microorganisms are common in all groups; 3333 were detected exclusively in the OSCC group, 59 in disease control group, and 238 in healthy control group (Figure 3a, Supplementary Table S3). 

Regarding the classes detected in the groups, a Venn diagram showed that 59 classes are common in all groups; 72 were detected exclusively in the OSCC group, 2 in the disease control group, and 4 in the healthy control group (Figure 3b, Supplementary Table S3).

The Shannon and inverse-Simpson biodiversity indices have been calculated to evaluate the intra-sample variability (Supplementary Figure S2a,b) [21,22]. Those indices are commonly used to quantify the human microbiome diversity [23,24]. The index values are homogeneous among patient groups, and it suggests a similar intra-sample variability among the patient groups.

To better study the internal differences of oral microbiota composition in the OSCC group, a subdivision of the same group into early and advanced stage was performed (Supplementary Figure S3).

We also compared the average read counts between the early and advanced OSCC patients. We did not find organisms whose average counts are significantly different among the two groups (Supplementary Table S4).

The Venn diagram showed that 3186 microorganisms are common in early and advanced groups (Supplementary Figure S4a, Table S5). In the early group, 873 microorganisms were detected, 53 in the early and disease control groups, 175 in the early and healthy control groups, and 14 were common in the early, disease and healthy control groups.

While in the advanced group, 1132 microorganisms were detected exclusively in this group, 65 in the advanced and disease control groups, 241 in the advanced and healthy control group, and 23 were common in the early, disease and healthy control groups.

Regarding the difference of classes among intra-groups, a Venn diagram showed that 137 are common in early and advanced groups (Supplementary Figure S4b, Table S5). In the early group, 15 classes were detected exclusively in this group, 1 in the early and healthy control group, and no classes were common in the early and disease control group. 

In the advanced group, 17 classes were detected exclusively in this group, 6 in the advanced and healthy control group, and only 1 in the advanced and disease control groups. 

## 4. Discussion 

The aim of this single center study was to investigate the salivary microbiota composition in patients affected by OSCC.

To the best of our knowledge, this is the first study that analyzed the salivary microbiota composition employing the ONT in OSCC patients. ONT is a new generation of sensing platform; it can analyze native DNA or RNA and sequence any length of fragment [25]. Indeed, one of the main advantages of ONT is the possibility to generate very long sequencing reads (up to 206.5 kb for unique reads) [26]. 

In the metagenomics study, short sequencing reads derived from traditional short-read technologies may not span complex genomic regions (e.g., repeats, transposons) resulting in fragmented, partial genomes and ambiguous assembly of closely related species. Differently, ONTs’ reads can achieve an unbiased assembly of complete, closed genomes and plasmids from clinical research and microbiome samples, improving the traditional gene-level shotgun metagenomic analysis [27].

Another ability of the ONT is to sequence native DNA and RNA without the requirement of the pre-amplification of published targets, enabling both to discover any genome present in the sample and eliminating possible bias that is PCR-related [28,29]. 

In the present study, the highly expressed genes in the OSCC group’s microbiota were related to *Prevotella*, in detail: *Prevotella melaninogenica*, *Prevotella intermedia* and *Prevotella jejuni*. These findings are partially in agreement with a recent systematic review that showed a prevalence of *Prevotella*, as well as *Fusobacterium* and *Bacteroidetes*, in patients affected by OSCC [19]. 

*Prevotella* is a Gram-negative anaerobic pathogen found in oral, vaginal, and gut microbiota [30]. In the oral cavity, *Prevotella* predominates in periodontal disease, periodontal abscesses and peri-implantitis [6]. 

In a study conducted by Granato et al. using Illumina MiSeq, *Prevotella* was also one of the most abundant microorganisms detected in salivary samples of patients affected by oral cancer. Moreover, in this study, the relative abundance of *Prevotella* was observed to correlate with local metastasis presence [31].

In particular, some studies suggested a possible correlation between *Prevotella melaninogenica* and oral carcinogenesis [32,33]. One study reported significantly higher levels of *Prevotella melaninogenica* in OSCC patients compared with the controls and a diagnostic sensitivity and specificity ≥ 80% [34].

With respect to *Prevotella intermedia*, its predominance was reported in patients affected by oral diseases, such as oral leukoplakia [35]. The same bacteria have been hypothesized to be involved in oral carcinogenesis producing volatile sulphur compounds (e.g., sulphuric acid), which promote the development and accumulation of genetic mutations [36].

With respect to *Prevotella jejuni*, in our knowledge, no data were reported about its implication in oral disease and carcinogenesis.

Beyond the *Prevotella*, in the present study, in the OSCC group high levels of *Chlamydiia*, *Tissierellia*, *Calothrix*, *Leotiomycetes*, *Firmicutes* and *Zetaproteobacteria* were detected. The most common genera detected in control group was *Saccharibacteria*.

*Chlamydia* infection has been mostly observed in pelvic inflammatory disease and it has been related to cervical cancer [37]. Regarding the oral cavity, *Chlamydia* was usually detected in patients affected by periodontitis [38]. No data about *Chlamydia* infection and OSCC onset was found.

Regarding *Tissierellia*, in the literature there are no relevant specific information about the association of these bacteria with any type of cancer or oral diseases; nevertheless, *Tissierellia* belongs to the phyla of *Firmicutes*, which is involved in carcinogenesis process in other districts, such as in colorectal cancer [19,39]. Regarding the oral cavity, several studies observed its prevalence in salivary microbiota of patients affected by OSCC [10,40,41]. Sawant et al. observed a prevalence of *Firmicutes* in tobacco chewers affected by OSCC [42].

Regarding *Calothrix*, no evidence in the literature was observed about their association with cancer or oral disease. Although *Calothrix* is a genus of *Cyanobacteria* and the latter has been reported to possess anti-HIV, anti-tuberculosis, anti-viral and anti-microbial activity [43,44]. Our literature review highlighted that only Hernandez et al. observed that *Cyanobacteria* was positively associated with hepatocellular carcinoma [45]. 

Evidence on *Leothiomycetes* is very limited. No data about *Leothiomycetes* infection and oral disease or OSCC onset was found. Only Liu et al. reported that *Leotiomycetes* were more abundant in patients with liver cirrhosis [46].

Zetaproteobacteria has been observed in various human body sites, including the oral cavity, skin, and vaginal tract [47]—but it is not reported to be implicated in carcinogenesis. However, *Zetaproteobacteria* is a class of *Proteobacteria*. *Proteobacteria* were described in a recent systematic review, together with *Firmicutes* and *Bacteroidetes*, as the most prevalent bacteria detected in the saliva of patients affected by OSCC [48].

*Saccharibacteria* was significantly less abundant in the OSCC group, in accordance with what has been previously reported [49]. *Saccharibacteria* are epibionts living on the surface of their host bacteria and are correlated with dysbiotic microbiomes during inflammatory diseases, including periodontitis [50]. Some studies observed that in periodontitis patients *Saccharibacteria* decreased inflammatory bone loss, serving a potential protective role in inflammatory damage [50,51]. 

In the last decade, the role of oral microbiome has been increasingly investigated [6,13,52]. Particular microorganisms have shown their capability to contribute to carcinogenesis (e.g., *Helicobacter pylori*) [7]. Regarding the potential role of oral microbiota in the oral carcinogenesis process, few studies have investigated the salivary microbiota composition in patients affected by OSCC compared to the healthy control [10,49,53,54]. 

Even if it is difficult to identify a causal relationship between oral microbiota and carcinogenesis, some oral bacteria, especially periodontal pathogens, have been shown to have carcinogenic potential [55]. These periodontopathic bacteria are involved in oral carcinogenesis through several different mechanisms, such as the development of chronic pro-inflammatory processes, direct anti-apoptotic action, and the production of carcinogenic metabolites [56,57]. However, a clear association between oral microbiome composition and oral carcinogenesis is still lacking. The latter may be related to the complexity of oral microbiome as well as to the design of the studies [19]. The oral microbiome is a complex dynamic ecological community composed of very distinct niches adhering to different surfaces of the oral cavity [13]. Additionally, there are still no studies on the oral microbiome of patients affected by potentially malignant oral disorders that successively developed OSCC. Furthermore, the salivary microbiome analysis method is very heterogeneous among the studies [19].

This study possesses some limitations, possibly derived from the small sample size, mainly due to the high sequencing cost, which limits the statistical power of the study. Since this is a preliminary observational pilot study, our future perspective is to continue recruiting patients and analyzing salivary oral microbiome to confirm this preliminary data. 

However, despite the small study group, the test group is representative with respect to worldwide epidemiology data. Indeed, among the 24 OSCC patients enrolled, most of the patients were males with a mean age of 65.5 years. These data agree with those reported in the literature, since OSCC globally affects more frequently males between the sixth and seventh decades of life [56]. The border of the tongue was the most affected site and there was homogeneity in the patient’s TNM stage distribution; even this datum was in line with the literature [56,57]. 

Although there were no statistically significant results, it seems that the most abundant microorganism detected may be related to periodontal infection. If these results are confirmed in future research, the latter may have considerable implications in the public health strategy. It is well established that periodic professional oral hygiene and lifestyle habits modifications may decrease the risk of periodontitis; such a prevention strategy could also prevent the risk of OSCC onset. 

Additionally, it is hoped that in the near future the costs for the microbiota analysis will be reduced, facilitating the analysis of more samples and therefore the possibility of having more accurate information on oral microbiota associated with OSCC. 

Moreover, our future perspective is to conduct a prospective study to be able to compare the oral microbiome of those healthy and OPMD patients who develop OSCC, before and after malignant transformation.

## 5. Conclusions

Undoubtedly, recognizing a specific oral microbiota associated with OSCC could favor understanding OSCC pathogenesis, primary and secondary prevention, and the follow-up of patients affected by OSCC. If the association between periodontal pathogens and OSCC will be confirmed by future studies, the control of periodontal status would assume a central role in the follow-up visit to reduce the localized inflammatory insult and slow down the progression of potentially malignant oral lesions and oral cancer. Since this is the first study using ONT, we hope that in the near future studies confirm our results employing the same methodology.

## Figures and Tables

**Table 1:** Characteristics of patients enrolled in the study.

Characteristics	Patients Affected by OSCC	Patients OSCC Free
	24 (77.4)	7 (22.6)
***Gender***		
Male	13 (54.2)	5 (71.4)
Female	11 (45.8)	2 (28.6)
***Demographic data***		
Median age	64.5	55
Q1–Q3	53.5–79.7	30–76
Mean age ± SD	65.5 ± 13.9	51.4 ± 19.2
***Risk factors***		
Tobacco smoking	4 (16.6)	0
Tobacco smoking and alcohol consumption	3 (12.5)	0
Former smoking	3 (12.5)	0
**Characteristics of patients affected by OSCC (n. 24)**		
***OSCC anatomical site***		
Border of the tongue	10 (41.7)	
Buccal mucosa	6 (25)	
Floor of the mouth	3 (12.5)	
Lower gingiva	3 (12.5)	
Upper gingiva	1 (4.1)	
Hard palate	1 (4.1)	
***TNM staging***		
Stage I	7 (29.2)	
Stage II	6 (25)	
Stage III	5 (20.8)	
Stage IV	6 (25)	

## Data Availability

Data is contained within the article or Supplementary Material.

## Supplementary Materials

The following supporting information can be downloaded at https://www.mdpi.com/article/10.3390/cancers15174211/s1.
