# Supplementary material for: No Clear Clustering Dysbiosis from Salivary Microbiota Analysis by Long Sequencing Reads in Patients Affected by Oral Squamous Cell Carcinoma: A Single Center Study
Source: Cancers (Basel). 2023 Aug 22;15(17):4211. doi: 10.3390/cancers15174211 (PMC10486367; doi:10.3390/cancers15174211)

cancers

Supplementary Materials

Supplementary Figure 1. Absolute frequencies of organism classes over patients.

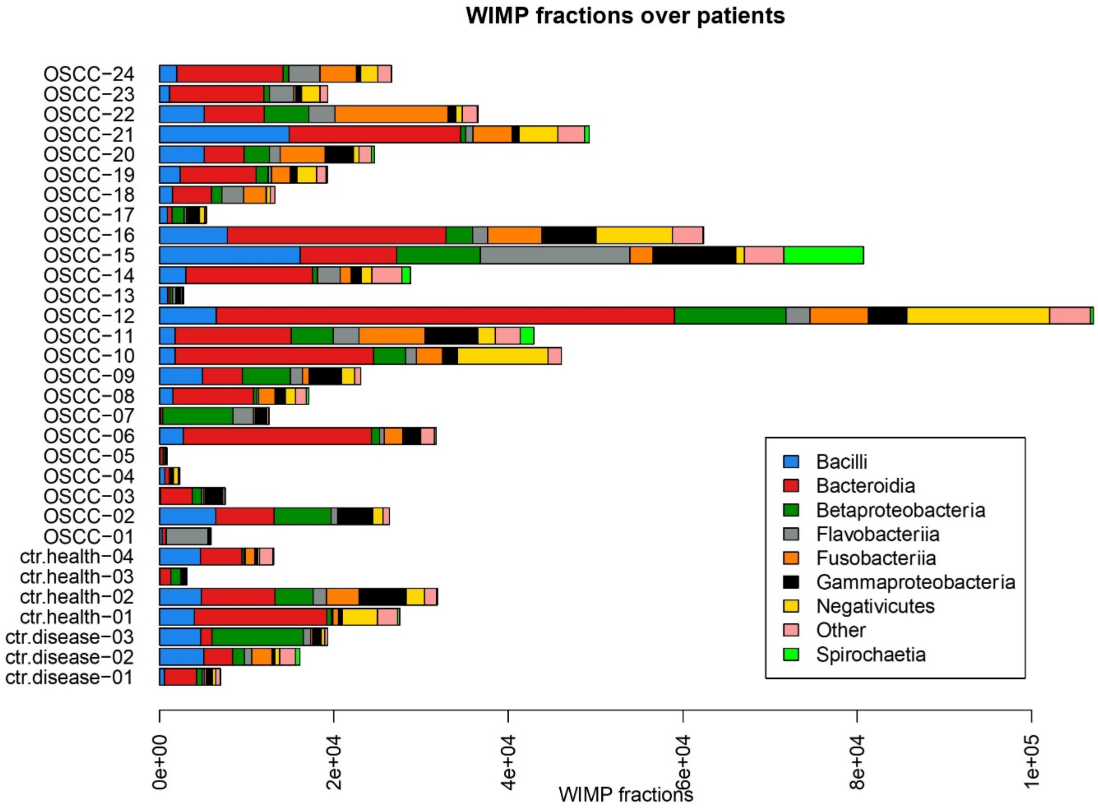

Supplementary Table 1. Comparison of average absolute frequences between test and control groups (control =disease + health).

	organism	average.test	average.control	logFC	pval	adj.pval
1	Chlamydiia	1.46	0.29	2.35	0.0024	0.30
2	Tissierellia	109.62	21.29	2.36	0.0100	0.31
3	Calothrix	1.17	0.00	Inf	0.0100	0.31
4	Leotiomycetes	0.29	0.00	Inf	0.0130	0.31
5	Firmicutes	22.79	7.71	1.56	0.0150	0.31
6	Zetaproteobacteria	0.29	0.00	Inf	0.0150	0.31
7	Nostoc	2.50	0.71	1.81	0.0200	0.31
8	Chondrocystis	0.29	0.00	Inf	0.0240	0.31
9	Bacteroidetes/Chlorobi	0.67	0.14	2.22	0.0270	0.31
10	Saccharibacteria	0.00	0.71	−Inf	0.0270	0.31
11	Saccharomycetes	1.54	0.29	2.43	0.0280	0.31
12	Dictyoglomia	0.29	0.00	Inf	0.0320	0.33
13	Insecta	5.42	1.57	1.79	0.0380	0.35
14	Synergistia	0.54	0.00	Inf	0.0410	0.35
15	Thermoplasmata	0.25	0.00	Inf	0.0430	0.35
16	Pseudanabaena	0.33	0.00	Inf	0.0480	0.35
17	Bacteroidetes	27.00	12.00	1.17	0.0610	0.35
18	Rippkaea	0.25	0.00	Inf	0.0630	0.35
19	Clostridia	351.71	145.86	1.27	0.0640	0.35
20	Chitinophagia	15.71	7.29	1.11	0.0710	0.35
21	Methanococci	0.83	0.29	1.54	0.0730	0.35
22	Sphingobacteriia	24.54	11.29	1.12	0.0740	0.35
23	Thermococci	0.88	0.29	1.61	0.0740	0.35
24	Methanomicrobia	2.08	0.86	1.28	0.0760	0.35
25	Gastropoda	0.38	0.00	Inf	0.0780	0.35
26	Anthozoa	0.38	0.00	Inf	0.0790	0.35
27	Methanobacteria	1.50	0.57	1.39	0.0810	0.35
28	Fusobacteriia	2711.25	1202.43	1.17	0.0830	0.35
29	Mollicutes	40.38	15.71	1.36	0.0830	0.35
30	Eurotiomycetes	0.29	0.00	Inf	0.0860	0.35
31	Bacteroidia	10587.96	5404.29	0.97	0.1000	0.36
32	Erysipelotrichia	11.50	4.71	1.29	0.1000	0.36
33	Ktedonobacteria	0.42	0.00	Inf	0.1100	0.36
34	Candidatus Saccharimonia	106.75	28.57	1.90	0.1200	0.36
35	Crinalium	0.38	0.00	Inf	0.1200	0.36
36	Holophagae	0.21	0.00	Inf	0.1200	0.36
37	Cyanobium	0.21	0.00	Inf	0.1200	0.36
38	Bacillus	5.75	2.14	1.42	0.1300	0.36
39	Microcystis	0.58	0.00	Inf	0.1300	0.36
40	Trichodesmium	0.25	0.00	Inf	0.1300	0.36
41	Candidatus Thermofonsia	0.21	0.00	Inf	0.1300	0.36

Supplementary Figures 2a. Organism diversity indices over patients.

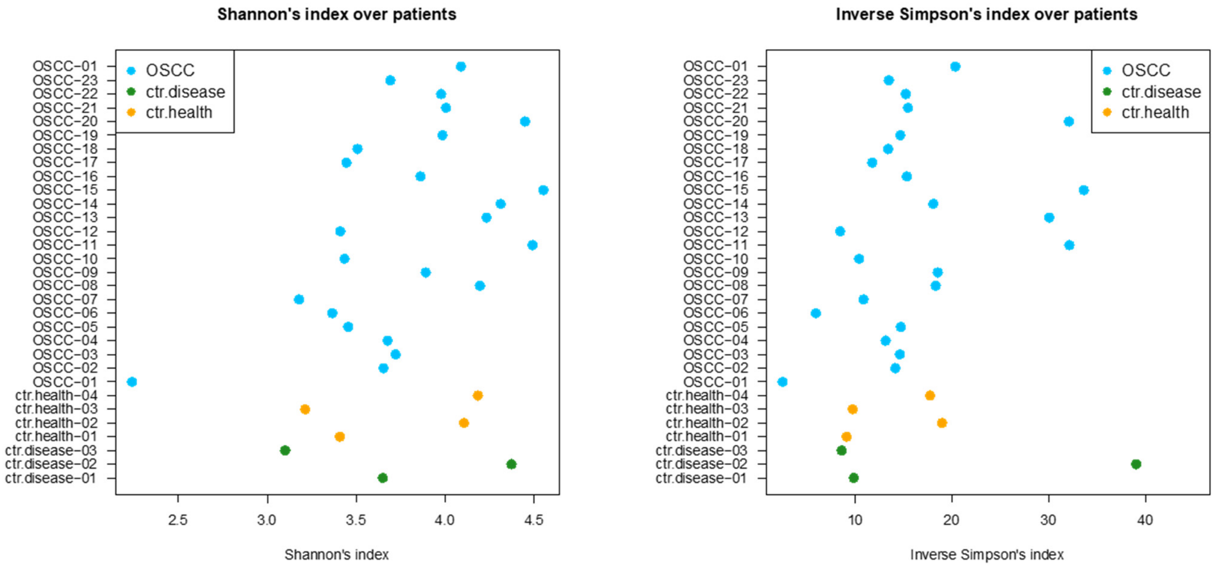

Supplementary Figures 2b. Class diversity indices over patients.

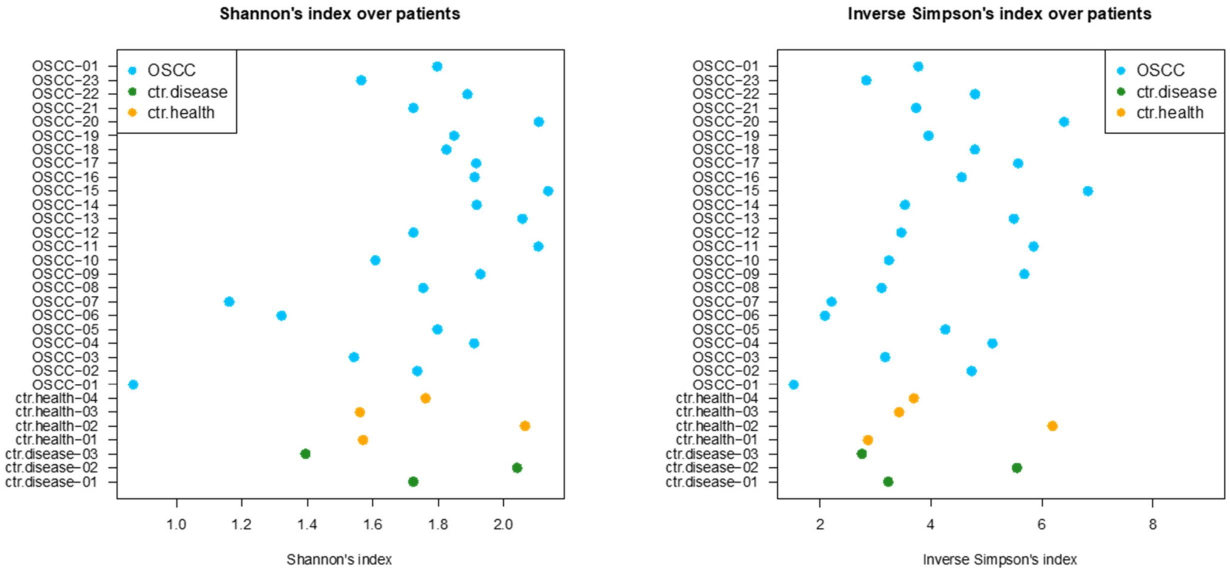

Supplementary Figure 3. Hierarchical clustering over patients.

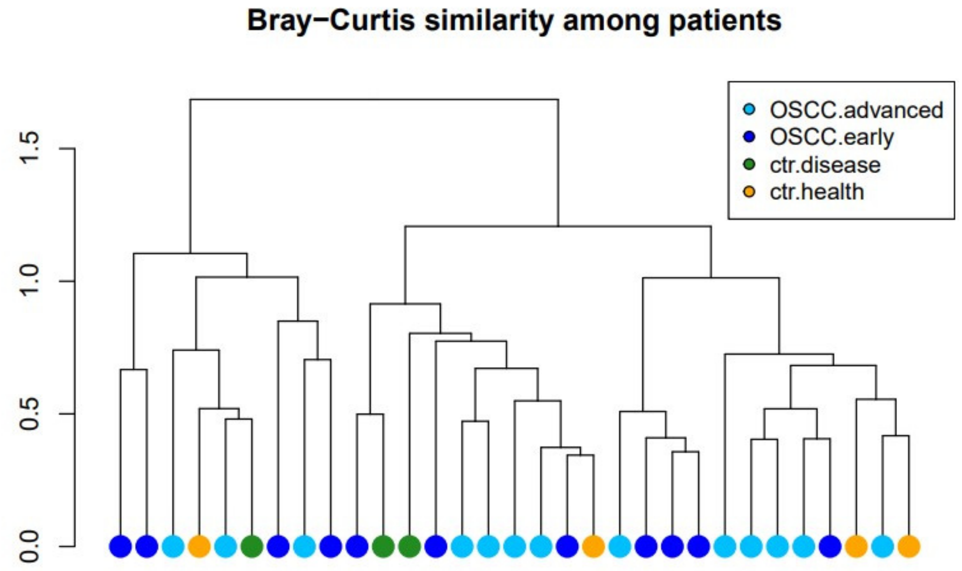

Supplementary Figure 4a. Organism overlaps among groups.

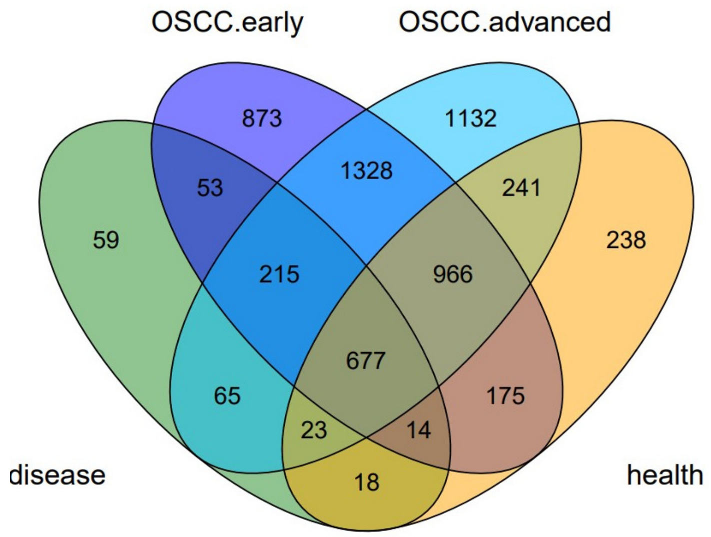

Supplementary Figure 4b. Class overlaps among groups.

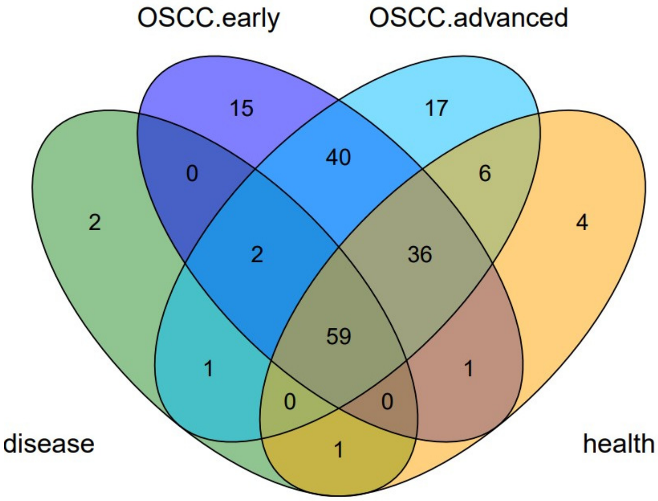

Supplement: Supplementary file 1 [file cancers-15-04211-s001.zip › cancers-2505863-supplementary Figures.pdf]
